# Toxic Metal Content in Mexican Propolis: A Human Health Risk Assessment

**DOI:** 10.3390/foods15071128

**Published:** 2026-03-25

**Authors:** Ana Paola Balderrama-Carmona, Leticia García-Rico, Ana Lilia López-Duarte, Melissa Valenzuela-Rincón, Luis Gerardo Ochoa-Balderrama, Guadalupe González-Ochoa, Lilian Karen Flores-Mendoza, Mario Eduardo Almada-Ortiz, Edgar Felipe Moran-Palacio, María Mercedes Meza-Montenegro

**Affiliations:** 1 Departamento de Ciencias Químico-Biológicas y Agropecuarias, Universidad de Sonora, Navojoa 85890, Sonora, Mexico; a221213766@unison.mx (M.V.-R.); guadalupe.gonzalezochoa@unison.mx (G.G.-O.); lilian.flores@unison.mx (L.K.F.-M.); mario.almada@unison.mx (M.E.A.-O.); edgar.moran@unison.mx (E.F.M.-P.); 2 Laboratorio de Contaminantes Metálicos, Coordinación de Ciencia de los Alimentos, Centro de Investigación en Alimentación y Desarrollo, Hermosillo 83340, Sonora, Mexico; lgarciar@ciad.mx (L.G.-R.); alopez@ciad.mx (A.L.L.-D.); 3 Departamento de Física, Matemáticas e Ingenierías, Universidad de Sonora, Navojoa 85890, Sonora, Mexico; a224230127@unison.mx; 4 Departamento de Biotecnología y Ciencias Alimentarias, Instituto Tecnológico de Sonora, Ciudad Obregón 85000, Sonora, Mexico; mmeza@itson.edu.mx

**Keywords:** risk assessment, chemometric analysis, arsenic, lead, manganese, cadmium, ICP-OES

## Abstract

In the present study, arsenic, cadmium, lead, and manganese contamination in propolis was assessed. A total of 12 raw propolis samples were analysed using atomic emission spectrometry with inductively coupled plasma (ICP-OES) for each of the metals. The analyses revealed that the samples were contaminated with each of the following metals: arsenic (ranging from <0.21 to 0.4 ppm), cadmium (<0.008 to 0.123 ppm), lead (0.580 to 117.01 ppm), and manganese (4.22 to 47.57 ppm). Lead levels exceeded the acceptable limits for consumption. A chemometric characterization was performed, looking at the correlation between elemental contamination, showing high correlation between cadmium and manganese levels (r = 0.80). Furthermore, the samples with the highest contamination levels were in areas with high industrial and agricultural activity. A risk assessment for toxic metals indicated a risk associated with lead. While the amount of lead present in the propolis samples was deemed to pose a low carcinogenic risk, it is noteworthy that the levels of the other metals detected do not pose any discernible health risk associated with the consumption of propolis. This conclusion is crucial for understanding the safety profile of propolis as a dietary supplement or natural product.

## 1. Introduction

Northwestern Mexico is known for its agricultural, industrial, and mining activities. However, there is growing concern about the environment quality due to the reported presence of toxic compounds [1], including, arsenic (As), cadmium (Cd), and lead (Pb) in water [2,3,4], soil [5] and food [1], and persistent minerals such as manganese (Mn) [6]. Arsenic (As) is a metalloid that tends to accumulate in the body, is classified as a group 1 carcinogen to humans, and chronic exposure to low to medium concentrations lead to a wide range non-carcinogenic effects as dermal, cardiovascular and endocrine disorders and it is also linked to lung, kidney, skin and nerve cancers [7]. Cd and Pb are heavy metals that are toxic, bioaccumulative, and persistent. Elevated levels of Cd can result in conditions such as Itai-Itai disease, as well as kidney problems, hypertension, and cancer [8,9]. Pb also impacts kidney function and exhibits neurotoxic effects, particularly in children, where it can decrease the intelligence quotient [10]. Mn is an essential element for humans; however, at high consumption levels, it can induce neurotoxic effects [11]. Therefore, these toxic metals represent significant risk to environmental and human health, emphasizing the need to conduct constant monitoring of contamination levels to mitigate public health risks [12].

Arsenic deserves particular attention due to its high toxicity and its widespread occurrence in environmental matrices. Unlike some other metals, arsenic can be readily mobilized in soil and groundwater, increasing the likelihood of human exposure through drinking water and food products [7]. Chronic arsenic exposure has been extensively documented as a major public health concern and is associated with carcinogenic and systemic effects, including damage to the skin, respiratory system, kidneys, and nervous system. Because of these characteristics and its strong association with long-term health risks, arsenic is often considered a key element in environmental risk assessments, making its monitoring particularly important in regions characterized by intensive agricultural, industrial, and mining activities [13].

Likewise, bee products such as honey, wax, and propolis are a viable option to be used as bioindicators of environmental pollution [13,14,15,16,17]. During foraging activities, bees leave their hives to collect nectar, pollen, and water from the environment where they are exposed to environmental changes as a result of the increase in toxic compounds present in plants, water, and soil. Bees are highly sensitive to these changes and can accumulate pollutants and incorporating these substances in their bodies and bee products [18,19]. In addition, if the bees are exposed to adverse environmental conditions, their production of honey may decrease; this is due to the close link between propolis and hive health, because bees protect the hive by covering it with propolis, protecting it from toxic metals [13,15,20].

Propolis is a natural substance of resinous gummy consistency, elaborated mostly by bees, especially of species *Apis mellifera* [13,15]. The gathering process for the elaboration of propolis begins when the worker bees collect the resin from plants; due to the variety in their diet, the bees acquire various waxes, resins, and plant exudates [21], which are mixed with various enzymes [22]. To obtain propolis, beekeepers can use specialized collection methods, such as propolis traps placed in beehives [23]. Propolis contains various bioactive chemical compounds with antimicrobial, antioxidant, anti-inflammatory, and anti-proliferative activity [24,25], undergoes after a propolis grinding process, is obtaining a fine powder that is more manageable and convenient for use in various health and wellness products, including, but not limited to, tinctures, capsules, creams, ointments, and tablets [26].

In Mexico, the demand for propolis has increased significantly in recent years, attracting interest from the pharmaceutical, cosmetic, food and health industries [27] since propolis can be used to develop new treatments against infectious diseases of various microorganisms [28], lipid metabolism [29], glycemic control [30,31], and oxidative stress [32]. Propolis-based treatments are typically administered orally; however, there is no standardized guidance regarding the appropriate dosage or frequency of intake. The recommended daily dosage of natural propolis is typically suggested to fall within the range of 900 to 1000 mg of natural propolis by day in adults [33,34,35]. Consumers may take propolis in different formulations, such as tincture [29] or capsules [32], with recommended frequencies ranging from every 15 days to every 3 months [30,32]. Propolis products intended for the treatments mentioned above are packaged in suitable containers, labelled under local and national regulations, and distributed through natural food stores, supplement shops, pharmacies, and online platforms [36].

Several major propolis-producing countries (Argentina, Cuba, and El Salvador) [37] already have regulations for its production and processing. This legislation includes maximum permissible limits of Pb, Cd, and As. Mexican propolis standards do not regulate the levels of toxic compounds in (NOM-003-SAG/GAN-2017) [38]. This may result in possible health risks for people who consume propolis regularly. The objectives of this study were: (1) to determine the levels of As, Cd, Pb, and Mn in samples of propolis obtained from northwestern Mexico, (2) to perform a chemometric analysis to find out a correlation between the metal composition and geographical origin, and (3) to evaluate the potential human health risks associated with exposure to toxic metals contained in Mexican propolis.

## 2. Materials and Methods

### 2.1. Study Area

**Figure 1:**
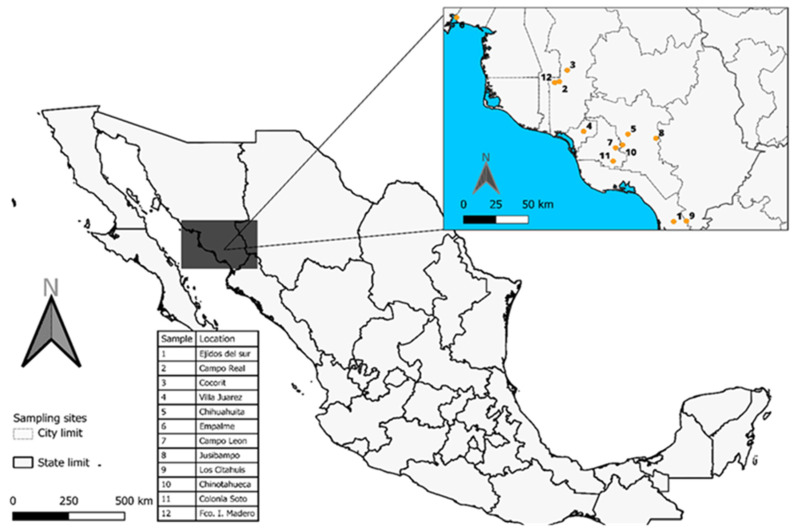
Geographic location of the sampling sites in agricultural Valleys of Northwest Mexico. The main map presents the national territory, highlighting the northwestern region where the study area is located.

The samples were collected from different beehives in the south of the State of Sonora (Figure 1), according to Mexican norm criteria (NOM-003-SAG/GAN-2017). Between 100 and 300 g of propolis was collected during a sampling period from May to October 2023. The propolis was scraped directly from the hive frames with a plastic spatula. The propolis samples were placed in new airtight bags covered with aluminum foil to protect them from light, labelled for later identification. Then, they were transported to the Biochemistry and Toxicology Laboratory of the University of Sonora, Navojoa Campus, where they were stored in a freezer at −20 °C until their analysis.

### 2.2. Characteristics of the Samples of Propolis

Different tones of brown samples (Table 1). These variations in the physical appearance of the propolis samples were observed may be due to the distinct species of plants.

### 2.3. Sample Preparation

Samples were homogenized, and subsamples were stored in sealed Petri dishes at −20 °C until analysis. Metal concentrations were determined by Inductively Coupled Plasma Optical Emission Spectrometry (ICP-OES; Thermo Fisher Scientific, Waltham, MA, USA) following microwave-assisted acid digestion. Approximately 0.2 g of propolis was digested in a microwave vessel (CEM Corporation, Matthews, NC, USA) with 4 mL HNO_3_ (70%, Fermont, Monterrey, Mexico), 1 mL H_2_O_2_ (29–32%, Meyer, CDMX, Mexico), and 4 mL ultrapure water. After predigestion (1 h), samples were digested in a Mars Xpress microwave system (CEM Corporation, Matthews, NC, USA) at 210 °C for 35 min. Digests were filtered through Whatman No. 4 filter paper, diluted to 25 mL with ultrapure water, and analyzed by ICP-OES. Analytical wavelengths (nm) for As, Cd, Pb, and Mn were 189.042, 214.438, 220.353, and 257.610, respectively. Instrumental conditions included RF power of 1150 W, plasma gas flow of 12 L min^−1^ Ar and nebulizer gas flow of 0.5 L min^−1^; external calibration was performed using multi-element standards with linear ranges appropriate for each element (R^2^ ≥ 0.999). The mixture was predigested for 1 h and subsequently mineralized using a microwave digestion system (Mars Xpress, CEM Corporation, Matthews, NC, USA) at 210 °C for 35 min.

### 2.4. Quality Control

All labware was washed with 20% nitric acid and rinsed with double-distilled water [39]. For quality control, blanks, triplicate samples, and the certified reference material SRM 1566b Oyster Tissue (National Institute of Standards and Technology, Gaithersburg, MD, USA) were analyzed together with the samples to verify the certified trace element concentrations [40]. The accuracy (expressed as recovery), precision, limits of detection (LOD), and limits of quantification (LOQ) for the analyzed metals were calculated according to Eurachem guide procedures (Table 1). Precision was expressed as relative standard deviation (RSD, %), obtained from replicate measurements, with an acceptance criterion of RSD ≤ 15%. Accuracy was evaluated using the certified reference material SRM 1566b, with acceptable recovery values ranging from 80 to 120%.

### 2.5. Chemometric Characterization of Propolis Based on Metal Content

#### 2.5.1. Correlation Graph

A correlation matrix was established between metals using the R package ‘corrplot’ version 0.92. A correlation coefficient (r value) was designated as high when r > 0.5, and moderate when the values were in the range of 0.3 to 0.5 [41].

#### 2.5.2. Principal Component Analysis (PCA)

Principal component analysis (PCA) was conducted to examine correlations among metals, specifically lead (Pb), manganese (Mn), arsenic (As), and cadmium (Cd). PCA was conducted using the XLSTAT software (version 2023.1.6.1410).

The cluster analysis used R package “clusters” version 2.1.6. The distance matrix was calculated using the Euclidean method. The clustering was then performed using Ward’s method [41].

### 2.6. Risk Assessment

The average daily dose (ADD) was performed according to the following equation:

(1) ADD = C × IngR × EF × ED/BW × AT,

where C is the metals concentration in propolis samples (ppm); IngR is the consumption of propolis per day (1000 mg [33] daily equivalent to 0.001 kg day^−1^); EF is the frequency of exposure (for 3 months, equivalent to 90 days year^−1^); ED is the duration of exposure (40 years) taking into account a single treatment over the course of an adult’s life, assuming an adult’s span of exposure of 40 years; BW, is the average weight of the adult person (75 kg) [42], and AT, is the average time (365 days multiplicate for years of exposure).

To assess non-carcinogenic human health risk from the consumption of propolis contaminated by metals, the hazard quotient (HQ) was estimated using the following equation:

(2) HQ = ADD/RfD, 

where ADD is the daily metal intake and RfD is the oral reference dose. The RfD (mg kgbw^−1^ day^−1^) for As is 0.0003, for Cd it is 0.001, for Mn is 0.14, and for Pb it is 0.004 [43]. If HQ > 1, the value could pose a potential risk.

Taking into account that both As [44] and Cd [45] are considered carcinogenic, and Pb is possibly carcinogenic to humans [46]; the carcinogenic risk (CR) for these metals was calculated using the following equation:

(3) CR = ADD/SF,

The ADD (average daily dose) is computed using the average time (AT) for a life expectancy of 75.5 years [47]. The cancer slope factor (SF) measures the risk of cancer increases with exposure. For As [48,49], the SF (mg kgbw^−1^ day^−1^) is 1.5, for Cd it is 0.0061 [49], and for Pb it is 0.0085 [48,49]. If CR > 1 × 10^−6^, it is considered a carcinogenic risk; a value ≥ 1 × 10^−3^ is viewed as a substantial risk; a value ≥1 × 10^−4^ < 1 × 10^−3^ is considered a moderate carcinogenic risk; a value ≥1 × 10^−5^ < 0.0001 is considered a low carcinogenic risk; a value < 1 × 10^−6^ is considered a very low carcinogenic risk [50].

## 3. Results

### 3.1. Quality Parameters of the Analytical Method for the Determination of Metals by ICP-OES

Table 2 shows the results obtained for the quality parameters of the method and performance of the ICP-OES in propolis [19,39,51,52]. In the present research, precision, expressed as RSD (%), was less than 15%, while accuracy (expressed as recovery %) ranged between 85% and 120% [40]. The detection limits were between 0.0028 and 0.076 ppm, for Cd and As and Mn, respectively. The limits of quantification were between 0.008 to 0.20 ppm, for Cd and As and Mn, respectively.

### 3.2. Concentration of Heavy Metals in Propolis

**Figure 2:**
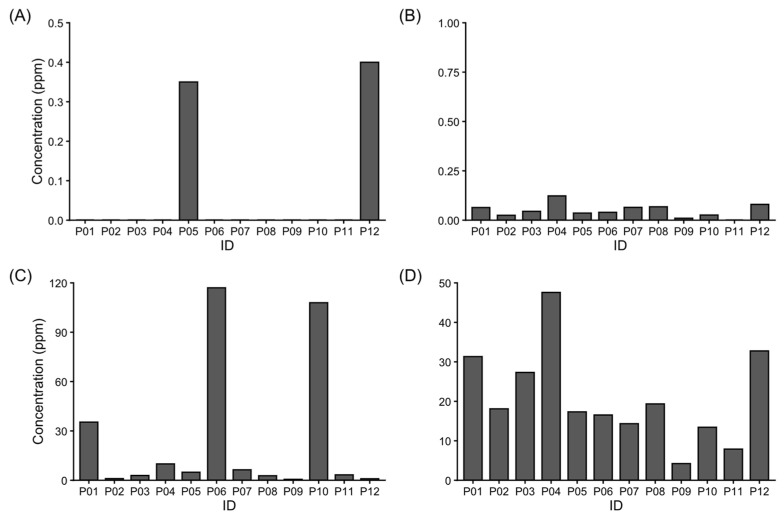
Metal content concentration in crude propolis: (**A**) As, (**B**) Cd, (**C**) Pb, and (**D**) Mn.

The concentrations of metals detected in the analyzed propolis samples (*n* = 12) are shown in Figure 2, where 77% of the samples analyzed had metal concentrations above the threshold values established in international guidelines for propolis, indicating a considerable presence of environmental contamination in the study area [53].

### 3.3. Chemometric Characterization of Propolis Based on Metal Content: Multivariate Analysis

**Figure 3:**
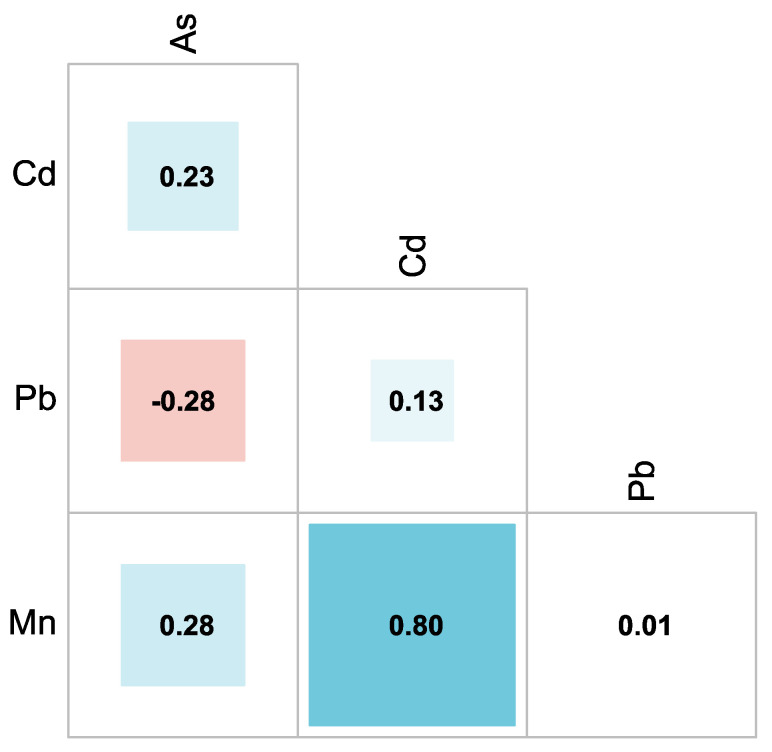
Correlation matrix of metal concentrations.

Figure 3 shows a correlation matrix with strong positive correlation between Cd and Mn (r = 0.80), suggesting similar environmental characteristics or potential common sources. In contrast, the remaining metal pairs exhibit weak correlations, indicating largely independent distribution patterns among the analyzed elements.

**Figure 4:**
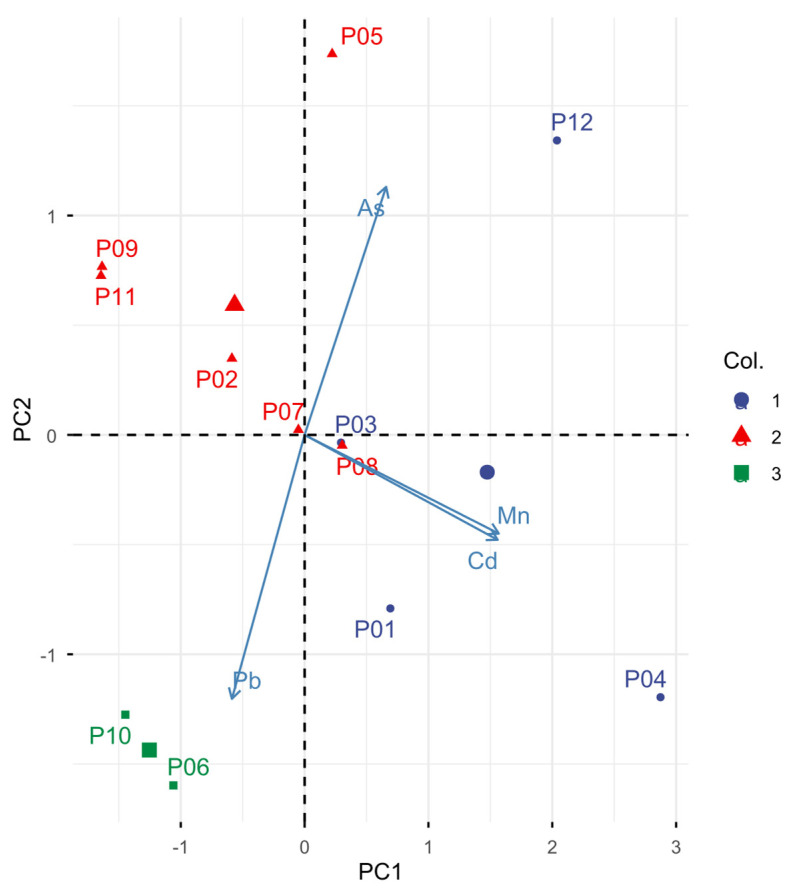
Principal component analysis biplot.

In Figure 4, the first two principal components explain 78% of the total variance in the dataset, with PC1 accounting for 50.1% and PC2 for 27.9%. PC1 is mainly associated with Cd and Mn, while PC2 shows negative loadings for Pb and As.

**Figure 5:**
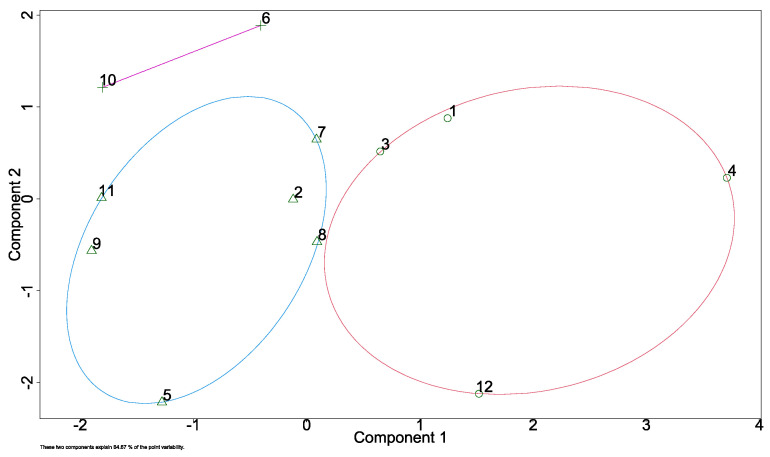
Main components cluster plot.

In Figure 5, in the blue cluster, four samples are similar in their toxicity level (P2, P5, P7, P8, P 9, and P11), while the red cluster represents four samples (samples P1, P3, P4 and P12). P06 and P10 are shown as outliers. From Figure 5, we can assume that P06 and P10 association are related to high Pb influence.

**Figure 6:**
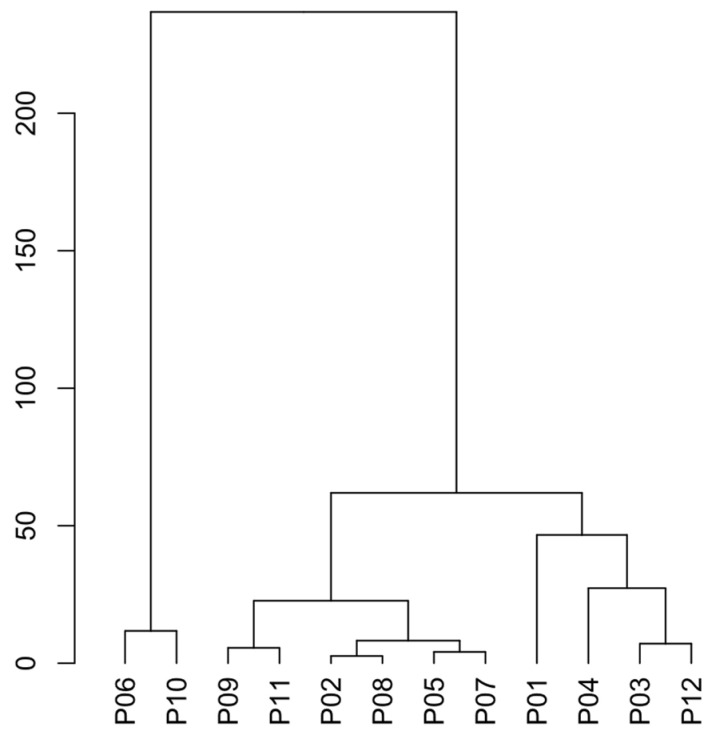
Ward’s dendrogram.

In Figure 6, for data normalization and cluster analysis, Ward’s dendrogram [51] was used to confirm the results, the similarities between the samples are clear. Samples P06 and P10 exhibit atypical behavior compared to others.

### 3.4. Risk Assessment Results

**Figure 7:**
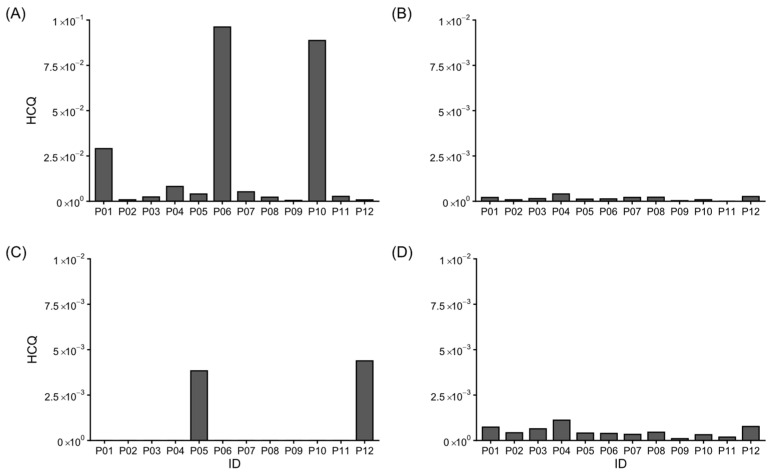
Non-carcinogenic hazard quotient: (**A**) Pb, (**B**) Cd, (**C**) As, and (**D**) Mn.

The HQ in the studied localities (Figure 7) revealed heavy metal exposure, with a notable exception in the regions of Chinotahueca and Empalme.

**Figure 8:**
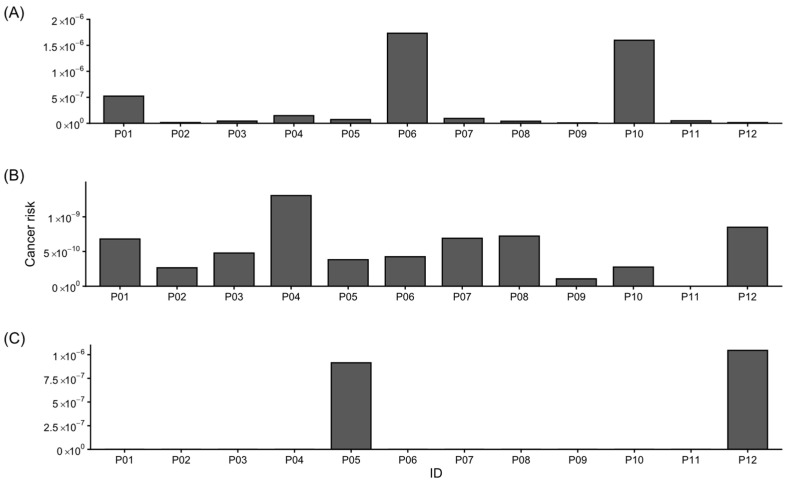
Carcinogenic hazard quotient: (**A**) Pb, (**B**) Cd, (**C**) As.

Figure 8 presents calculated cancer risk associated with Pb (A), Cd (B), and As (C) across the sampling sites. Pb-related risk shows higher values at stations P06 and P10 compared with the remaining locations. Cd-associated cancer risk exhibits moderate variability among sites, whereas the highest As-related risk values were observed at stations P05 and P12.

## 4. Discussion

Regulation (EU) 2020/640 from the European Food Safety Authority (EFSA) specifies that, in propolis, the maximum permissible level (MPL) for Cd should not exceed 0.1 ppm, for Pb 1 ppm, and As should be absent in the samples (Figure 3) [54]. In Latin America, the Argentine standard IRAM-INTA 15935 [55] establishes limits of <10 ppm for Pb and <2 ppm for As, whereas the Cuban regulation NRAG-1135 (M32.1)-94 [56] sets limits of 2 ppm for Pb and 1 ppm for As. Additionally, EFSA indicates that daily Mn intake should not exceed 3 mg [53].Toxic metals in propolis samples are attributed to anthropogenic activities in the areas near to hives, such as industrial and mining activities [16] or the use and application of chemical products and fertilizers in irrigation canals and agricultural areas [19,57]. They can also be contaminated by naturally occurring problems such as gas emissions, volcanic eruptions, and forest fires due to global warming and the leaching process of chemicals that cannot be degraded [51].

Concentrations of As were found in two propolis samples: (16.67% of total samples), P05 and P012 (Figure 2). This metal is found in high concentrations in groundwater [58] globally. In the area studied, the presence of As has been reported in groundwater [59,60,61] and soil [5,62]. The concentrations of As found in this study were not within the permissible or safe limit range according to EFSA regulations. Previous research conducted by Vakhonina et al. [63] on propolis showed that the concentration of As ranged from 0 to 1.04 ppm. In our study, the maximum concentration of As was 0.35 ppm, value which is between the range values previously reported (0.087–1.238 ppm) by Roman et al. [64]. The As pathways in propolis depend on factors such as climate and seasons, refs. [16,18] for example, the presence of moisture plays a crucial role in reducing the resuspension of dust and the generation of particulate matter that might carry the metalloid. Specifically, in agricultural soils in these study regions, the primary sources of contamination are pesticides that contain arsenate [60].

Cd was detected in 92% of propolis samples (Figure 2), with only one exception (sample P11). The concentrations of Cd in our samples ranged from <0.008 to 0.123 ppm. The literature shows similar concentrations [13,15,16,19,39,51]. However, Formicki et al. [65] obtained Cd concentrations in propolis samples ranging from 1.48 to 10.77 ppm, significantly higher than our results. On the other hand, Tutun et al. [39] reported Cd levels below detection limits (˂0.067 µg kg^−1^), using ICP-OES. The sample P04, collected from the Villa Juárez area, exceeded the MPL, the rest of the propolis samples had Cd concentrations below the MPL. The study area is characterized by high agricultural activities and the use of phosphate fertilizers, organic manures, and soil acidification which could elevate the Cd concentration [66] in this region. Propolis serves as an effective bioindicator of contamination. There are some studies which have reported higher levels of Cd in propolis compared with honey [64]. However, in pollen, contamination levels are similar [67].

The presence of Pb was detected in 100% of the propolis samples (Figure 1). Numerous studies have reported the presence of Pb in propolis [3,15,16,38,51,63,64,67,68,69,70,71,72,73]. Similar results have been reported in propolis from Brazil [13], Serbia [51], Croatia [16], Austria [72], Argentina [74], and Spain [70]. However, in México, propolis samples produced in the state of Hidalgo did not show detectable levels of Pb [67], but in the state of Yucatan, concentrations of Pb in propolis-based tints were found to be below 2.8 µg L^−1^ [38]. Our results showed concentrations of Pb that significantly exceed those reported in the central and southern regions of Mexico. Notably, the propolis samples (P06 and P10) which were collected from Empalme and Chinotahueca, respectively, showed Pb levels of 117.01 and 107.93 ppm, respectively. Empalme is an urban port, and it is worth mentioning that the sampling site in this town was located less than eight hundred meters away from a road and a couple of kilometers from the port. Several studies have reported elevated levels of Pb in soil adjacent to highways, attributed to the use of Pb in gasoline [16,19,51], to old paint on highways, and wheel weights [75]. Since 2021, Pb has been permanently removed from gasoline [76]. Chinotahueca is in the heart of the Mayo Valley which is surrounded by agricultural fields dedicated to chickpea, wheat, and safflower plantations. Agricultural activity has consistently been associated with elevated levels of Pb [10,16,19,51,65]. Pb in the environment is also attributed to the accumulation of Pb in batteries, electronic equipment and mining activities. Alcici’s study [71] in Brazilian propolis reports significant differences between the concentrations of Pb in painted and unpainted bee boxes, indicating that paint is a determining factor in the increase in Pb in propolis.

Mn is an essential mineral for human cardiometabolic health [11,77,78]. High concentrations of this metal, however, can be correlated with high concentrations of other metals like Pb and Hg [78]. Hence, it can be associated with issues in child neurodevelopment. Furthermore, a connection between exposure to Mn and metabolic syndrome has been established [77]. Mn has also been associated with neurotoxicity when its levels exceed 1 ppm [11]. In the present study, Mn was present in 100% of our samples, exceeding the EFSA and MPL values set by international standards. The Mn concentration was in the range of 4.22–47.57 mg kg^−1^, and the highest concentrations were found in Huatabampo, Benito Juárez, and Francisco I. Madero regions. Other studies have documented maximum Mn concentrations of 28.0 [39], 27.1 [70], 8.232 [16], and 6.51 [51] ppm. These concentrations were lower than those reported in our current study. In our study region (south Sonora), the quality of tap water is a current concern for the population. This mineral causes significant sediment deposits and darkens the tap water. This environmental route is the most understandable for the introduction of Mn into the beehive, as bees drink water to regulate their body temperature [79]. Mn contamination in propolis is attributed to the natural presence of Mn in the environment [80].

According to the chemometric analysis conducted, there is no statistically significant relationship between the content of toxic metals and the different geographical areas analyzed (*p* ≥ 0.5). This can be due to the large area of sampling, which is dominated by a few economic activities such as agriculture, mining, and industry, and lacks urban areas. In contrast, with other research studies, which have found significance between metal concentrations and sampling sites [13,57]. Hodel et al. [15] reported similar correlation samples. P06 and P10 represent the highest values on the positive side of PC2, due to their high concentrations of Pb. Sample P04 represents a high concentration of Mn and is at the positive extreme of PC1. Finger et al. [13] reported an association between Cd and Pb, and this association was also found by Formicki et al. [57], suggesting a common source. In this investigation, however, Cd was found to be associated with Mn. Samples P10 and P06 are seen grouped in an atypical cluster 3 (Figure 4) due to their unusual Pb contamination compared to other samples. These data reflect the high concentration of Pb in the sampled geographical areas, which, as mentioned earlier, may be due to their proximity to roads [75], high agricultural activity [10,19] and hive paint [71].

In these specific areas, a marked increase in Pb exposure was observed, suggesting a notable elevation in comparison with the low levels found in other areas. This heightened Pb exposure is potentially linked to anthropogenic origin. Manzanares-Rivera [81] indicates that, in Sonora, there is an increase in kidney diseases, due mainly to environmental causes; they acknowledge that, even at low levels of Pb exposure, it is associated with kidney failure. Even though exposure to As is also related to chronic kidney diseases [82], it is not possible to carry out cumulative risk assessment because the health effects of the evaluated metals occur in different organs of the body.

A carcinogenic risk for Pb exceeding 1 × 10^−6^ is observed in samples of propolis from Ejidos del Sur, Empalme, and Chinotahueca. However, it is important to note that this level of risk is considered non-concerning according to the 7 levels of risk based on the Delphi method [48]. All the other metals evaluated exhibit completely acceptable CR levels. It is worth noting that for therapeutic use, propolis is consumed in the form of extracts or tinctures, which are certified to contain much lower amounts of Pb than raw propolis [68]. Mineral content has been reported to be significantly lower in tinctures than in raw propolis [83]. Orsi et al. [14] detailed that the reduction in metals in propolis after processing into extracts ranges from 24 to 100%. It is necessary to regulate the content of toxic metals in Mexican legislation to enhance the quality of propolis and prevent harm to consumers’ health since there are raw propolis treatments available in Mexico in capsule form [83].

The potential risks of underestimation propolis via dermal exposure from its use in creams and cosmetic products, has not been considered [84]. It is important to highlight that raw propolis plays a vital role as a bioindicator of contamination. It is therefore worth noting that the northwest area of Mexico may be contaminated with toxic metals. It is recommended that future studies investigate the source of these metals in propolis. Furthermore, it is suggested that comprehensive health risk assessments be carried out by collecting various environmental samples, such as food, air, soil, water, and bee products for each of the toxic metals under study.

## 5. Conclusions

This study is the first research to quantify metals in propolis samples from Sonora, Mexico. Propolis collected in the northwest region of Mexico was found to contain As, Cd, Pb, and Mn in 16.67%, 92%, 100%, and 100% of the samples, respectively. There was no significant correlation observed among the different geographical zones of the sampling sites. The amount of Pb present in the propolis poses a low carcinogenic risk, and the other metals do not pose any health risk for the consumption of propolis.

## Figures and Tables

**Table 1:** The harvested propolis obtained were different tones according to the sampling site.

ID	Color	Region
P01	Black	Ejidos del Sur, Huatabampo
P02	Dark brown	Rancho Real, Cajeme
P03	Black	Cócorit, Cajeme
P04	Black	Villa Juárez, Benito Juárez
P05	Brown	Chihuahuita, Navojoa
P06	Greenish brown	Empalme, Nuevo Guaymas
P07	Black	Campo León, Etchojoa
P08	Amber	Jusibampo, Navojoa
P09	Brown	Los Citahuis, Álamos
P10	Amber	Chinotahueca, Navojoa
P11	Light brown	Colonia Soto, Etchojoa
P12	Dark brown	Fco. I. Madero, Cajeme

**Table 2:** Quality parameters of the ICP-OES method for the determination of metals.

Metal	Recovery	RSD	LOD	LOQ
	(%) *	(%) *	(ppm)	
As	85.00	0.70	0.076	0.210
Cd	90.14	0.15	0.0028	0.008
Pb	94.90	2.60	0.010	0.045
Mn	97.27	0.05	0.076	0.210

* Acceptance criteria: accuracy (as recovery) 80–120%; precision (as RSD) ≤ 15%.

## Data Availability

The original contributions presented in the study are included in the article. Further inquiries can be directed to the corresponding authors.
